# *Clostridium butyricum* Induces the Production and Glycosylation of Mucins in HT-29 Cells

**DOI:** 10.3389/fcimb.2021.668766

**Published:** 2021-06-17

**Authors:** Qi Lili, Lu Xiaohui, Mao Haiguang, Wang Jinbo

**Affiliations:** ^1^ School of Biological and Chemical Engineering, Ningbo Tech University, Ningbo, China; ^2^ Research Department, Ningbo Biomart Lifetech Co.Ltd, Ningbo, China

**Keywords:** *C. butyricum*, HT-29 cells, mucins, glycosylation, adhesion

## Abstract

*C. butyricum* is a common gut commensal bacterium, which has many positive functions in human intestine. In this study, we investigated the effects of monosaccharide and its derivatives on the adhesion of *C. butyricum* to the mucus of HT-29 cells. RNA interference was performed to assess the roles of MUC2 and glycan in the adhesion of *C. butyricum* to HT-29 cells. The effects of *C. butyricum* on the glycosylation of mucins were assayed with fluorescence microscope. The expression levels of mucins and glycotransferases were also determined. The results showed that *C. butyricum* could adhere to the mucins secreted by HT-29 cells. Several kinds of monosaccharides inhibited the adhesion of *C. butyricum* to HT-29 cells, which suggested that the mucus glycan was the attaching sites of this bacterium. Knockdown of MUC2, FUT2 or GALNT7 significantly decreased the numbers of the bacteria adhering to HT-29 cells. When colonizing on the surface of HT-29 cells, *C. butyricum* could increase the production of mucins, promote the expression of glycotransferase, and induce the glycosylation of mucins. These results demonstrated that the glycan of mucus played important roles in the adhesion of *C. butyricum* to HT-29 cells. This study indicates for the first time that *C. butyricum* possesses the ability to modulate the glycosylation profile of mucus secreted by HT-29 cells. These findings contribute to understanding the mechanism of interaction between colonic epithelial cells and commensal bacteria.

## Introduction

Human gastrointestinal tract (GIT) is inhabited by trillions of bacteria with more than 1000 identified species, with a density of 10^11^ bacterial cells per gram wet weight in the colon contents (Rehman et al., 2011; Parker et al., 2020). These intestinal bacteria involve in many critical physiological functions related to host health (Kaur et al., 2020; Reyes et al., 2020; Xu et al., 2020). In healthy individuals, the composition of intestinal microbiota usually remains in constant equilibrium for a long period of time (Guo et al., 2020; Leon-Coria et al., 2020). The gastrointestinal epithelium is covered by a layer of mucus, which consists of at least nine kinds of mucins. The mucus layer could supply inhabitants and energy resource for the intestinal commensal microbes. Moreover, the mucus is also the first barrier protecting the gut from being invaded by the bacteria. Mucus is critical for the intestinal health since disruption of this boundary will probably result in bacterial penetration of mucus barrier and induce intestinal inflammation (Liu et al., 2020; Sharma et al., 2020; Son et al., 2020). It has been reported that gut bacteria could colonize the intestinal mucus layer *in vivo* and adhere to HT-29 cell *in vitro* (Altamimi et al., 2016; Engevik et al., 2019).

Most mucins are found to express in human colon, including Mucin1, Mucin2, Mucin3, Mucin4 and Mucin5AC, of which MUC2 (Mucin 2) is the major gel-forming mucin (Pelaseyed and Hansson, 2020). Mucins are glycosylated proteins, which are characterized by a high content of carbohydrates such as N-acetylgalactosamine (GalNAc), N-acetylglucosamine (GlcNAc), galactose (Gal), fucose (Fuc) and sialic acid. Carbohydrates constitute up to 80% of the mucin mass. A series of glycosyltransferases are responsible for the addition of the monosaccharide or its derivatives to the sugar chain of mucins (Arike et al., 2017; Pelaseyed and Hansson, 2020). Probiotics such as *Lactobacillus* and *Bifidobaterium* have been demonstrated to increase the expression and secretion of MUC2 and MUC3 in intestinal epithelial cells (Mack et al., 2003; Subramani et al., 2015). Protein O-glycosylation is initiated by GalNAc-Ts, which could add GalNAc to the Ser or Thr residues of mucins (Arike and Hansson, 2016; Arike et al., 2017). GALNT7 (N-acetylgalactosaminyltransferase 7) is the most abundantly expressed GalNAc transferase in colon. C2GnT2 is highly expressed in the mucin producing tissues such as colon and stomach, which could catalyze the transfer of GlcNAc to the C6 carbon of the initial GalNAc. ST3Gal family are α-2,3-STs, which catalyze the transfer of sialic acid residues to galactopyranosyl (Gal) residue (Arike et al., 2017). ST3Gal3 is involved in catalyzing the transfer of sialic acid residues onto sugar chains of mucins. FUT2 (Fucosyltransferase 2) is the only identified enzyme to catalyze the addition of terminal α1,2-fucose residues to Gal of the glycans (Carvalho et al., 2010; Lee et al., 2010; Arike et al., 2017). Previous studies have indicated that intestinal commensal bacteria could stimulate the expression of several glycosyltranferases, which are responsible for catalyzing the transfer of carbohydrates to mucins (Wrzosek et al., 2013; Engevik et al., 2019). The oligosaccharide structure of mucins may play important roles in the adhesion of gut bacteria to the intestinal epithelial cells (Altamimi et al., 2016).

*C. butyricum* is a strictly anaerobic bacterium, which is a common gut commensal bacterium. Some non-toxigenic strains have positive functions to prevent intestinal diseases such as ulcerative colitis, inflammation and colonic cancer (Gao et al., 2012a; Gao et al., 2012b; Gao et al., 2012c). *C. butyricum* MIYAIRI 588 probiotic strain has been clinically approved for the treatment of diarrhea and other intestinal diseases in Japan (Hagihara et al., 2019). This bacterium could colonize human intestinal tract, adhere to the epithelium, produce short-chain fatty acid and modulate host intestinal immunity (Hagihara et al, 2020). In addition, HT-29 cell line harbors a glycosylated mucus layer, which can be used as an *in vitro* model for the adhesion of bacteria to human intestinal epithelium (Altamimi et al., 2016). However, no studies have shown that whether *C. butyricum* could induce the production and glycosylation of mucins in HT-29 cells.

In this study, we investigated the effects of monosaccharide and its derivatives on the adhesion of *C. butyricum* to the mucus of HT-29 cells. Knockdown of MUC2, GALNT7 and FUT2 was performed to determine the roles of MUC2 and glycan in the adhesion of *C.butyricum*. Furthermore, the glycosylation of mucins was detected and then the expression levels of glycotransferases were analyzed. This study is among the first to identify *C. butyricum* modulates mucus production and induces the glycosylation of mucins.

## Materials and Methods

### Bacterial Strains and Culture Conditions

*C. butyricum* MIYAIRI II588 was obtained from Miyarisan Pharmaceutical Co., Ltd. (Tokyo, Japan). *C. butyricum* was anaerobically cultured in RCM (Reinforced Clostridium Medium) medium at 37°C for overnight (about 16 hours). The strains were subcultured at 37°C for 4 h as required.

### Cell Culture

Colon adenocarcinoma cell line HT-29 was obtained from the Cell Bank of the Chinese Academy of Sciences (Shanghai, China). The cells were grown in Dulbecco’s modified Eagle’s medium supplemented with 10% fetal bovine serum, 100 unit/mL penicillin and 100 μg/mL streptomycin at 37°C in a 5% CO_2_ atmosphere in a humidified incubator. At the day prior to treatment with *C. butyricum*, the media were replaced by fresh media containing no antibiotics.

HEK293 cell line was also obtained from the Cell Bank of the Chinese Academy of Sciences (Shanghai, China). The cells were cultured in DMEM with 10% FBS, penicillin-streptomycin solution (penicillin 100 U/mL, streptomycin 0.1 mg/mL) at 37°C in a 5% CO_2_ in a cell incubator. The passage time of HEK293 cells was 80-90% confluence, and the passage ratio was 1:3 ~ 1:6.

### Preparation of Monosaccharides and the Derivatives

D(+)galactose, fucose, sialyl acid and N-Acetylglucosamine(NAG) were dissolved in PBS and microfiltered (0.2 μm sterile disc) then kept at 4°C till they were used for the experiment. The concentration of stock solutions was 10% w/v.

### Preparation and Labeling of Bacteria

*C. butyricum* MIYAIRI II588 were subcultured on 2×YTG (yeast extract 10 g, tryptone 16 g, and glycerol 5 g, NaCl 5 g per liter) plates overnight at 37°C and subsequently brought to log phase growth in RCM. After washing three times with normal saline, the bacteria pellets were collected by centrifugation at 2500 rpm for 5 min and resuspended in 2.5 μM SYTO9 in 0.01M PBS, and then incubated for 15 min in the dark. The excess SYTO9 was removed by washing three times with normal saline. For the adhesion assay, OD600 of *C. butyricum* resuspended in PBS was adjusted to 0.5(equivalent to 4×10^8^ CFU/mL).

### Bacterial Adhesion Assays

For adhesion assays, HT-29 cells were seeded in 96-well culture plates at a concentration of 4 × 10^4^ cells per well. After reaching 90% confluence, pre-warmed (37°C) monosaccharides or its derivative stock solutions were added to each well to obtain 1~3% (w/v) final concentration. Each monosaccharide or derivative was prepared in triplicate. 100μL SYTO9 labeled *C. butyricum* suspension was added per well and incubated for 1h in the dark. The bacteria suspension was then aspirated and washed three times with PBS. Fluorescence intensity was measured with a Synergy HT Multi-Detection Microplate Reader (BioTek^®^) using a 485/20 nm excitation filter and a 528/20 nm emission wavelength emission filter. Fluorescence intensity was measured for determination of the amount of binding bacteria. The results from the adhesion assay are presented as means from three independent experiments.

### Lectin Staining and Microscopy

After treatment with *C. butyricum* for 12 h, HT-29 cells were washed three times with normal saline. The cells then were incubated for 1h with FITC labeled lectin solution(1:1000dilution), which containing the α-1,2-fucose specific lectin ulex europaeus agglutinin (UEA) and GlcNAc specific lectin wheat germ agglutinin (WGA). After stained with the lectins, HT-29 cells were washed with pre-warmed PBS for three times. Fluorescence microscopic analyses were performed with a Nikon TE2000 inverted fluorescence microscope. ImageJ software was used for data acquisition and image analysis.

### RT-qPCR

Total RNA was extracted using the total RNA Kit (OMEGA Bio-Tek). RNA concentrations were determined at 260nm using NanoDrop 2000c UV-Vis Spectrophotometer (Thermo Fischer Scientific, Wilmington, DE) and purity was assessed by the A260/A280nm ratio. All procedures were according to the manufacturer’s instructions. RT-qPCR was performed using CFX Connect System (Bio-Rad, California, USA) with 2.5 μg of total RNA and 0.2 μM of each primer. The sequences of the primers corresponding to the glycosyltransferase and mucin genes are listed in **Table 1**. Thermal cycling conditions were as follows: 2min denaturation at 98°C, followed by 40 cycles at 98°C at 10s, 10s at 60°C, 30s at 68°C. Data were collected and analyzed using the CFX Manager software (Bio-Rad, California, USA). Cycle thresholds were normalized to GAPDH levels and fold changes were calculated to the normalized control of each gene. The relative mRNA levels of glycosyltransferase and MUCs were examined using the ΔΔCt method. Each sample was treated in triplicate to ensure statistical analysis significance.

**Table 1:** Primers used for qRT-PCR.

gene	Forward primer (5′-3′)	Reverse primer (3′-5′)
MUC3	AGGTGGGCATGGAAGTGTCT	CTGTAGGCCTGGGAAGTGTTGT
MUC2	TGTAGGCATCGCTCTTCTCA	GACACCATCTACCTCACCCG
C2GnT2	GCTTCCCGAGATTTCGTCCA	AACAGAGCCAGGCATCCACC
GALNT7	ATGCTAGTCGTCCTGAATCGC	GCGGTCCAGTGAGATCATGTC
ST3Gal3	GGTGGCAGTCGCAGGATTT	CATGCGAACGGTCTCATAGTAGTG
FUT2	TCAGATGCCTTTCTCCTTTCC	CTCCCACATGGCTTGAATCT
GAPDH	GAAGGTGAAGGTCGGAGTCAAC	CATCGCCCCACTTGATTTTGGA

### Western Blot Analysis

After reaching 85% confluence in a 6-well plate, HT-29 cells were exposed to 10^6^~10^8^ CFU/mL of *C. butyricum* for 12 h. Then the cells were washed with PBS for three times and lysed in 150 μL RIPA buffer according to the manufacturer’s instructions (Beyotime, Shanghai, China). Protein concentrations were measured with BCA Kit (Beyotime, Nantong, China).

50 μg of protein samples were separated by either 3–8% Tris-acetate gradient gels for MUC2 detection or 12.5% Tris-glycine gels for detection of other proteins. Then the protein samples were transferred to nitrocellulose membranes (Millipore, Burlington, MA,USA). After blocking with 5% skim milk in PBS-T, membranes were washed three times with PBS-T and then incubated in primary antibody at 4°C overnight, followed by incubation with secondary antibody at room temperature for 1 h. Protein bands were detected by ChemiDocXRS system (Bio-Rad, CA, USA) using ECL Western Blotting Substrate (Thermo Fisher, MA, USA). Anti-GALNT7 antibody (Abcam, ab113743), anti FUT2 antibody (Abcam, ab177239), anti-beta actin antibody (Abcam, ab227387), anti-MUC2 (Cell Signaling Technology, 88686S) and HRP-linked goat anti-mouse antibody (Cell SignalingTechnology, 33416S, MA, USA) were used for western blot analysis.

### RNA Interference

Three small interfering RNAs (siRNAs) targeting MUC2, GALNT7, fut2 and siRNA negative control (si-NC) were synthesized and purified by GenePharma (Shanghai, China). The sequences of these RNAs are shown in **Table 2**. The oligonucleotides were transfected into the cells using Lipofectamine 3000 Reagent (Invitrogen, Carlsbad, CA,USA) according to the manufacturer’s protocol. The transfection efficiency was detected by RT-qPCR.

**Table 2:** siRNA sequences used in this study.

Name	Sequence
si-NC-F	UUCUCCGAACGUGUCACGUTT
si-NC-R	ACGUGACACGUUCGGAGAATT
si-MUC2-1-F	GGUGGAGACACAGAAUUGATT
si-MUC2-1-R	UCAAUUCUGUGUCUCCACCTT
si-MUC2-2-F	GCCUCAACUACGAGAUCAATT
si-MUC2-2-R	UUGAUCUCGUAGUUGAGGCTT
si-MUC2-3-F	GUACGUUGGAGUUCUAUAATT
si-MUC2-3-R	UUAUAGAACUCCAACGUACTT
si-GALNT7-1-F	GGCAGUAUCUCACAUUUAATT
si-GALNT7-1-R	UUAAAUGUGAGAUACUGCCTT
si-GALNT7-2-F	GCCGCUUAUAGAUGUCAUATT
si-GALNT7-2-R	UAUGACAUCUAUAAGCGGCTT
si-GALNT7-3-F	GCAGUGUGGUGGCAAAUUATT
si-GALNT7-3-R	UAAUUUGCCACCACACUGCTT
si-FUT2-1-F	GUGCUAGCCUCAACAUCAATT
si-FUT2-1-R	UUGAUGUUGAGGCUAGCACTT
si-FUT2-2-F	GGGACUAUGUCCAUGUCAUTT
si-FUT2-2-R	AUGACAUGGACAUAGUCCCTT
si-FUT2-3-F	CCAUCUACCUGGCCAAUUATT
si-FUT2-3-R	UAAUUGGCCAGGUAGAUGGTT

### Statistical Analysis

For statistical analysis, a one way analysis of variance (ANOVA) was carried out for each comparison, followed by posthoc analysis to identify differences between specific factor levels using the Tukey “Honest Significant Difference” method. P-values less than 0.05 were regarded as statistically significant.

## Results

### Adhesion of *C. butyricum* to HT-29 Cells

**Figure 1:**
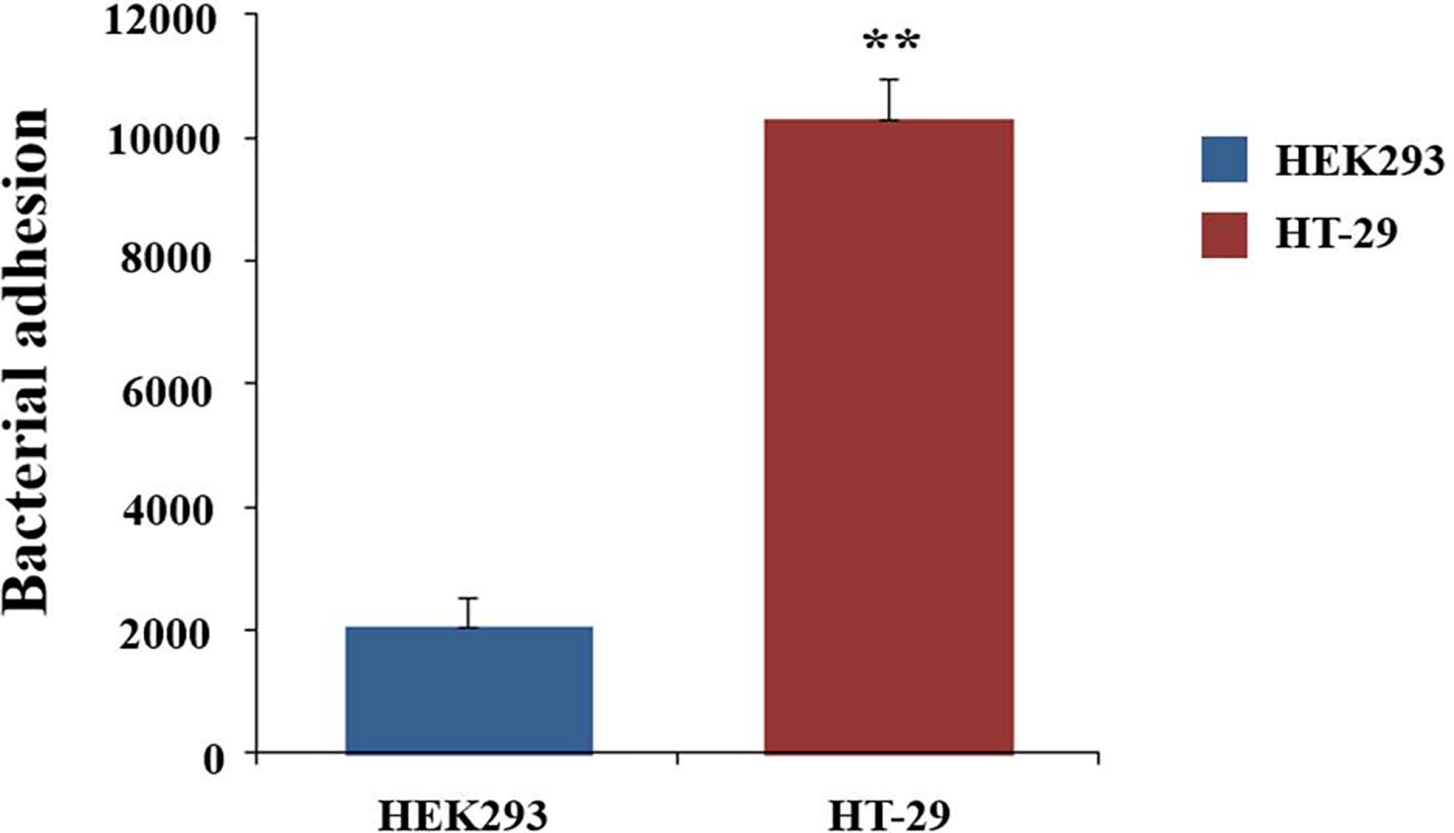
Adhesion of *C. butyricum* to HEK293 and HT-29 cell line. The live bacteria were fluorescently stained with SYTO9 and then incubated for 1h with HT-29 cells. The fluorescence intensity was measured with the microplate reader at 528nm. HEK293 cells was used as the control. Results are expressed as means and standard deviation of three independent experiments with **P < 0.01.

Adhesion to the mucus layer is considered to be an important process for colonization by probiotic microbes. To determine the adhesion capabilities of *C. butyricum*, the live bacteria were fluorescently stained with SYTO9 and then incubated with HT-29 cells for 1h. The fluorescence intensity was measured with the microplate reader. HEK293 cells, which did not express mucins, was used as the control. HT-29 cells exhibited 5.5-fold (*P*<0.01) higher fluorescence intensity compared to the HEK293 cells (**Figure 1**). This result indicated that *C. butyricum* could adhere to the surface of HT-29 cells. Thus, HT-29 could be used as the adhesion model of *C. butyricum*.

### Inhibition of *C. butyricum* Adhesion to HT-29 Cells by Monosaccharides and the Derivatives

**Figure 2:**
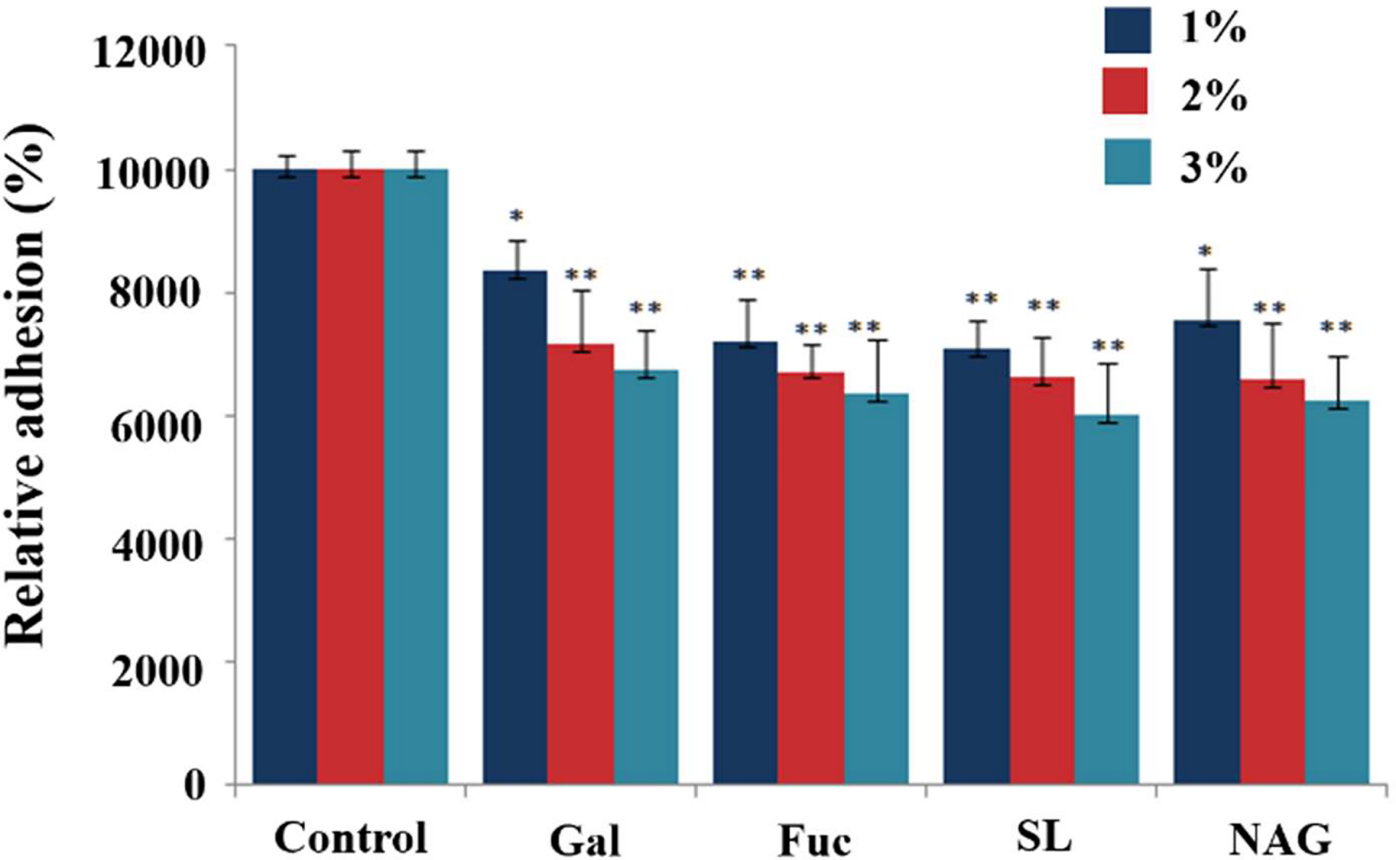
Effects of monosaccharide and its derivatives on the adhesion of *C. butyricum* to HT-29 cells. The live bacteria were fluorescently stained with SYTO9 and then incubated for 1h with HT-29 cells. The fluorescence intensity was measured with the microplate reader at 528nm. Results are expressed as means and standard deviation of three independent experiments with **P < 0.01 and *P < 0.05.

D(+)galactose, fucose, sialyl acid and NAG are the main terminal groups on the surface of digestive tract epithelial glycocalyx, which are speculated to be the candidate binding sites. To test the roles of glycan in the adhesion of the *C. butyricum* to the mucus, the bacteria was incubated with a range of protein-related monosaccharides or the derivatives prior to assaying the effects on adhesion. There was a significant reduction in the adhesion of *C. butyricum* to the mucus following incubation of the bacteria in any of the selected monosaccharides or derivatives(**Figure 2**). Compared to the control group, 1% and 2% of monosaccharides or derivatives resulted in 17-30% and 28-35% bacterial adhesion inhibition, respectively. When the cells were treated with 3% of monosaccharide or its derivatives, the bacterial adhesion decreased 33-40% (**Figure 2**) (*P*<0.01). There were no significant differences in the magnitude of inhibition of binding by the different monosaccharide or its derivatives at the same concentration.

### Knockdown of MUC2, GALNT7 and FUT2 Inhibits the Adhesion of *C. butyricum* to HT-29 Cells

**Figure 3:**
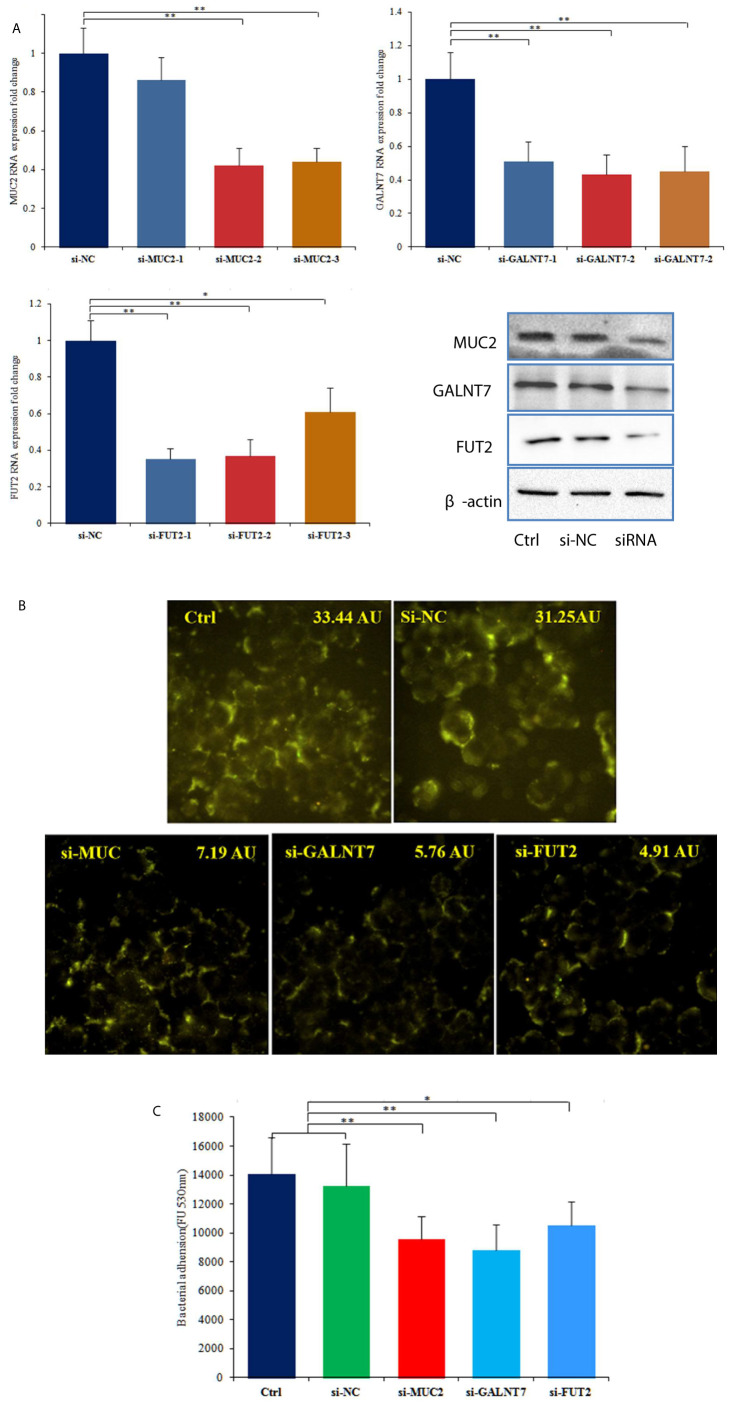
Knockdown of MUC2, GALNT7 or FUT2 decreased the adhesion of *C. butyricum* to HT-29 cells. n=3. **(A)** Knockdown efficiencies of MUC2, GALNT7 and FUT2 were examined by RT-qPCR in HT29 cells, and then western blot analysis was performed to verify the knockdown efficiencies. **(B)** After knockdown of MUC2, GALNT7 or FUT2, HT-29 cells were stained with FITC labeled agglutinin. The mean fluorescence intensity of each picture is 33.44, 31.25, 7.19, 5.76, 4.91 arbitrary units (AU) using ImageJ software. **(C)** Adhesion of *C. butyricum* to HT-29 cells was assayed by measure of the fluorescence intensity with the microplate reader at 528 nm. The si-MUC2-2, si-GALNT7-2 and si-FUT2-1 were used in this adhesion experiment. Results are expressed as means and standard deviation of three independent experiments with **P < 0.01 and *P < 0.05.

To further investigate the roles of MUC2 and GTs in the adhesion of *C. butyricum* to HT-29 cells, we transfected the cells with siRNAs targeting MUC2, GALNT7 and FUT2. RT-qPCR was used to examine the transfection efficiency, and the three siRNAs (si-MUC2-2, si-GALNT7-2 and si-FUT2-1) with highest transfection efficiency were used for further experiments (**Figure 3A**). 72 hours after transfected with si-MUC2-2, si-GALNT7-2 or si-FUT2-1, total protein of the cells was extracted and western blot analysis was performed. The results showed that siRNA transfection obviously reduced the protein levels of *MUC2, GALNT7 and FUT2* in HT-29 cells (**Figure 3A**). FITC labeled lectin staining results also indicated that siRNA transfection significantly attenuated the glycosylation of mucins (**Figure 3B**). Bacterial adhesion assay demonstrated that knockdown of MUC2, GALNT7 or FUT2 significantly inhibited the adhesion of *C. butyricum* to HT-29 cells (**Figure 3C**).

### *C. butyricum* Increases the Glycoprotein and Mucus Profiles of HT-29 Cells

**Figure 4:**
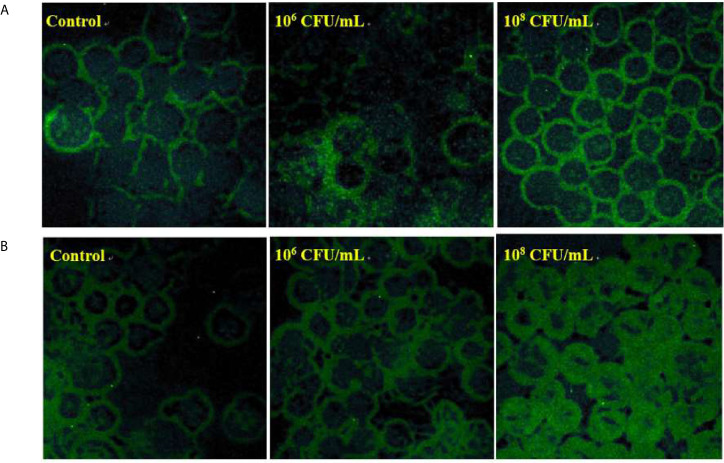
*C. butyricum* increases the glycoprotein and mucus profiles of HT-29 cells. n=3. **(A)** After pre-treatment with *C. butyricum* for 6h, HT-29 cells were stained with FITC labeled UEA. **(B)** After pre-treatment with *C. butyricum* for 6h, HT-29 cells were stained with FITC labeled WGA.

To examine whether *C. butyricum* influences the production of glycosylated mucins, FITC labeled lectin was used to visualize the glycan of mucins. After pre-treatment with *C. butyricum* for 6 hours, HT-29 cells were incubated with the α-1,2-fucose specific lectin ulex europaeus agglutinin (UEA) (**Figure 4A**) or with the GlcNAc specific lectin wheat germ agglutinin (WGA) (**Figure 4B**). In comparison with the control, the binding of WGA or UEA was significantly increased in the cells treated with 10^6^ or 10^8^ CFU/mL of *C. butyricum*.

As the increase in lectin staining caused by *C. butyricum* could either be related to an increase in the expression of mucin or glycosyltransferases involved in glycoprotein, we decided to study the influence of *C. butyricum* on the expression of these genes.

### *C. butyricum* Influences the Expression of Cellular Glycosyltransferases and Mucins in HT-29 Cells

**Figure 5:**
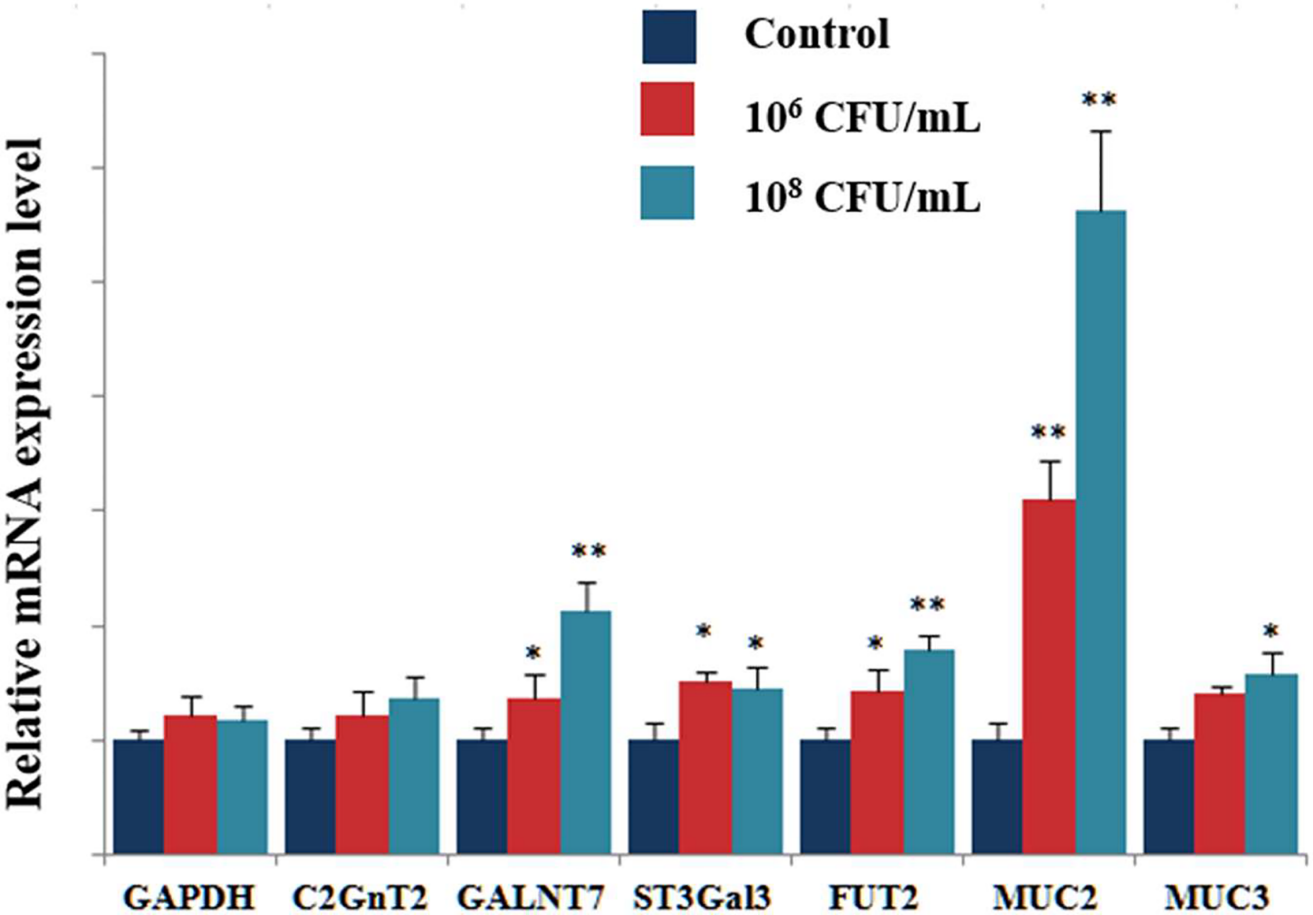
Effects of *C. butyricum* on the mRNA expression of GTs and mucins. RT-qPCR analysis was performed to determine the mRNA expression levels of GTs and mucins. Results are expressed as fold increase in GTs and Muc compared to untreated cell and normalized using GAPDH mRNA. Results are expressed as means and standard deviation of three independent experiments with **P < 0.01 and *P < 0.05.

We next tested the effects of treatment with *C. butyricum* (10^6^ or 10^8^CFU/mL) on the transcriptional expression of glycosyltransferases(GTs) by RT-qPCR. This study focused the analysis on the expression of the glycosyltransferases such as Core 2 β-1,6- N-acetylglucosaminyltransferase-2 (C2GnT2), N-acetylgalactosaminyltransferase 7 (GALNT7), β-galactoside-α-2,3- sialyltransferase3 (ST3Gal3), and Fucosyltransferase 2 (FUT2). **Figure 4** shows the relative mRNA expression levels for each glycosyltransferase gene. The results showed a significant increase in GALNT7, ST3Gal3 and FUT2 expression in HT-29 cells treated with 10^6^CFU/mL of *C. butyricum*. In the cells treated with 10^8^CFU/mL of *C. butyricum*, the relative expression of GALNT7, ST3Gal3 and FUT2 gene was 2.1, 1.5 and 1.7 times higher than that of control group. The relative expression of mucins was also significantly up-regulated. Compared with the control group, the mRNA expression of MUC2 showed a 5.6-fold increase (*P*<0.01), the mRNA expression of MUC3 showed a 1.6-fold increase (*P*<0.05) (**Figure 5**).

**Figure 6:**
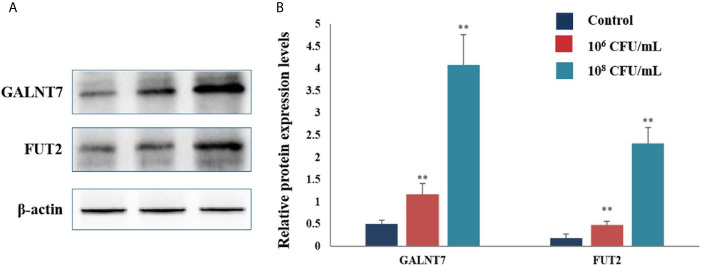
*C. butyricum* promoted the expression of GALNT7 and FUT2 of HT-29 cells. n=3. **(A)** Western blot analysis for GALNT7 and FUT2, normalized by β-actin. **(B)** Quantitative analysis for the densitometry of the proteins performed using ImageJ software. Results are expressed as means and standard deviation of three independent experiments with **P < 0.01.

The protein levels of GTs were also assayed by western blot analysis. Treatment with 10^6^ and 10^8^ CFU/mL of *C. butyricum* increased the expression levels of GALNT7 2.3- and 8.1-fold, respectively, compared with the control group. The levels of FUT2 were increased by 1.7- and 12.9-fold in the HT-29 cells treated with 10^6^ and 10^8^ CFU/mL of *C. butyricum*, respectively (**Figure 6**). These results indicated that *C. butyricum* could promote the expression of GTs and mucins of HT-29 cells in a dose-dependent manner.

## Discussion

Attachment to the digestive tract epithelium is very critical for the probiotics to play its physiological functions. The intestinal epithelial mucus are thought to be the colonizing place of probiotic bacteria (Sicard et al., 2017). One limitation of the study is that it was performed in HT-29 cell line, which was a cancer cell line, and not normal human tissue, but studies have shown that HT-29 cell line is able to produce and secrete mucins and form a mucus layer, which could be used as *in vitro* intestinal epithelium model. (Gagnon et al., 2013; Dudik et al., 2020). In this work, we explored the adhesion capability of *C. butyricum* to intestinal luminal epithelium by using HT-29 cells as an *in vitro* model. The results demonstrated that *C. butyricum* could adhere to HT-29 cells.

Previously, it has been dissected that O-Glycans on mucus are the main attachment sites of gut symbionts (Subramani et al., 2015; Ye et al., 2015). GalNAc usually is the initiating sugar in the O-glycans, which then extended by the addition of Gal, GlcNAc or GalNAc giving the Core1, Core2 and Core3 structures. The O-glycan chains are often terminated by sialic acid, GalNAc or fucose (Gagnon et al., 2013; Arike et al., 2017). Herein we demonstrated that galactose, fucose or sialic acid significantly inhibited the adhesion of *C. butyricum* to the HT-29 cells. The results suggested these monosaccharide or derivatives could interact with *C. butyricum*, and inhibit the recognition of the bacteria to mucus and decrease adherence of the bacteria to HT-29 cells. After transfection with siRNA targeting MUC2, GALNT7 or FUT2, we found the numbers of *C. butyricum* adhering to HT-29 cells decreased significantly. These results furtherly revealed that the glycans of mucus played key roles in the adhesion of *C. butyricum* to HT-29 cells. This research indicated that the glycan of mucus might be the candidate binding sites of *C. butyricum* to colonic tract luminal epithelium.

It has been reported that the probiotics such as *Bifidobacterium* and *Lactobacillus* could promote the secretion of mucins (Round et al., 2012; Wrzosek et al., 2013; Nishiyama et al., 2015; Arike et al., 2017). *C. butyricum* is an important commensal bacterium inhabiting human gut, which has been used as a clinical drug to cure intestinal inflammation in Japan (Dudik et al., 2020). In the present study, the fluorescence microscopy analysis revealed that co-culture with *C. butyricum* resulted in a marked elevation of the mucin secretion and/or glycosylation of the mucus in HT-29 cells. The precise mechanisms by which *C. butyricum* promotes the secretion and glycosylation of mucins and enhances the intestinal integrity remain to be elucidated. It has been reported that butyrate could upregulate the expression of MUC2 and MUC5A, increase the epithelial barrier function, induce the assembly of tight junctions and enhance the glycosylation of mucus in Caco-2 and HT-29 cells (Gaudier et al., 2004; Peng et al., 2009; Nielsen et al., 2018). Probiotics-derived short-chain fatty acids(SCFAs) could regulate epithelial barrier, promote mucus release, and induce differentiation of intestinal epithelial cells (Son et al., 2020). The bioactive components secreted by probiotic bacteria could also contribute to the expression of mucins and changes in O-glycosylation of mucins (Da Silva et al., 2014; Wang et al., 2014). *C. butyricum* can also secret lots of active bioactive factors, which play important roles in modulating intestinal functions (Morishita et al., 2021). Butyric acid is the main SCFA produced by *C. butyricum*, so we speculated the bacteria might stimulate the production of mucins through its metabolite butyric acid, and the bioactive factors secreted by *C. butyricum* might affect the production and glylcosylation of mucus. Further study should be carried out to discover the precise mechanisms by which *C.butyricum* increase the production and glycosylation of mucins.

Among the different human mucin genes, MUC2 and MUC3 are the predominant ileocolonic mucins. The MUC2 gene is expressed in goblet cells of large intestine, which is the major secreted mucin of the colon (Sicard et al., 2017). In addition, MUC2 form the barrier separating the bacteria from the colonocytes, but the transmembrane mucins form a second barrier at the level of the microvilli (Round et al., 2012). It has been indicated that probiotic bacteria could stimulate the expression of mucins (Wang et al., 2014). In this study, we found that exposure to *C. butyricum* resulted in significant up-regulation of the expression of secreted mucin MUC2 and transmembrane mucin MUC3.

Previously, it has been shown that commensal bacteria such as *Lactobacillus, Bifidobacterium* and *Bacteroides fragilis* could promote the gene expression of these glycotransferases (Caballero-Franco et al., 2007). Similar to these results, the present study demonstrated that exposure to *C. butyricum* significantly enhanced the expression of glycotransferases such as GALNT7, C2GnT2, ST3Gal3 and FUT2. These data indicated that *C. butyricum* induced glycosylation of mucins *via* up-regulation of the gene expression of glycotransferases.

In summary, the glycan play very critical roles in the adhesion of *C. butyricum* to HT-29 cell line. *C. butyricum* could stimulate the expression, secretion and glycosylation of mucins when colonizing on the surface of HT-29 cells. The present research suggests that *C. butyricum* is able to positively regulate intestinal epithelial barrier functions by inducing the glycosylation of mucins.

## Data Availability Statement

The original contributions presented in the study are included in the article/supplementary material. Further inquiries can be directed to the corresponding author.

## Author Contributions

Conceptualization: WJ and QL. Funding acquisition: WJ and QL. Formal analysis: WJ, LX, and QL. Supervision: WJ. Methodology: QL. Resources: LX and QL. Investigation: LX, MH, and QL. Validation: WJ. Data curation: WJ and QL. Writing original draft: QL. Writing review & editing: WJ. All authors contributed to the article and approved the submitted version.

## Funding

The authors declare that this study received funding from the National Natural Science Foundation of China and the Ningbo Science and Technology Bureau Project. The funder was not involved in the study design, collection, analysis, and interpretation of data, the writing of this article or the decision to submit it for publication.

## Conflict of Interest

Author LX was employed by the company Ningbo Biomart Lifetech Co.Ltd.

The remaining authors declare that the research was conducted in the absence of any commercial or financial relationships that could be construed as a potential conflict of interest.
