# Corrigendum: *Clostridium butyricum* induces the production and glycosylation of mucins in HT-29 cells

**DOI:** 10.3389/fcimb.2022.1038432

**Published:** 2022-09-29

**Authors:** Qi Lili, Lu Xiaohui, Mao Haiguang, Wang Jinbo

**Affiliations:** ^1^ School of Biological and Chemical Engineering, NingboTech University, Ningbo, China; ^2^ Research Department, Ningbo Biomart Lifetech Co.Ltd, Ningbo, China

**Keywords:** *C. butyricum*, HT-29 cells, mucins, glycosylation, adhesion

## Incorrect affiliation

In the published article, there was an error in affiliation(s) [1]. Instead of “[*School of Biological and Chemical Engineering, Ningbo Tech University, Ningbo, China*]”, it should be “[*School of Biological and Chemical Engineering, NingboTech University, Ningbo, China*]”.

The authors apologize for this error and state that this does not change the scientific conclusions of the article in any way.

## Publisher’s note

All claims expressed in this article are solely those of the authors and do not necessarily represent those of their affiliated organizations, or those of the publisher, the editors and the reviewers. Any product that may be evaluated in this article, or claim that may be made by its manufacturer, is not guaranteed or endorsed by the publisher.

